# An Antagomir to MicroRNA Let7f Promotes Neuroprotection in an Ischemic Stroke Model

**DOI:** 10.1371/journal.pone.0032662

**Published:** 2012-02-29

**Authors:** Amutha Selvamani, Pratheesh Sathyan, Rajesh C. Miranda, Farida Sohrabji

**Affiliations:** Neuroscience and Experimental Therapeutics, Texas A&M Health Science Center College of Medicine, College Station, Texas, United States of America; Charité-Universitätsmedizin Berlin, Germany

## Abstract

We previously showed that middle-aged female rats sustain a larger infarct following experimental stroke as compared to younger female rats, and paradoxically, estrogen treatment to the older group is neurotoxic. Plasma and brain insulin-like growth factor-1 (IGF-1) levels decrease with age. However, IGF-1 infusion following stroke, prevents estrogen neurotoxicity in middle-aged female rats. IGF1 is neuroprotective and well tolerated, but also has potentially undesirable side effects. We hypothesized that microRNAs (miRNAs) that target the IGF-1 signaling family for translation repression could be alternatively suppressed to promote IGF-1-like neuroprotection. Here, we report that two conserved IGF pathway regulatory microRNAs, Let7f and miR1, can be inhibited to mimic and even extend the neuroprotection afforded by IGF-1. Anti-mir1 treatment, as late as 4 hours following ischemia, significantly reduced cortical infarct volume in adult female rats, while anti-Let7 robustly reduced both cortical and striatal infarcts, and preserved sensorimotor function and interhemispheric neural integration. No neuroprotection was observed in animals treated with a brain specific miRNA unrelated to IGF-1 (anti-miR124). Remarkably, anti-Let7f was only effective in intact females but not males or ovariectomized females indicating that the gonadal steroid environment critically modifies miRNA action. Let7f is preferentially expressed in microglia in the ischemic hemisphere and confirmed in ex vivo cultures of microglia obtained from the cortex. While IGF-1 was undetectable in microglia harvested from the non-ischemic hemisphere, IGF-1 was expressed by microglia obtained from the ischemic cortex and was further elevated by anti-Let7f treatment. Collectively these data support a novel miRNA-based therapeutic strategy for neuroprotection following stroke.

## Introduction

The risk of ischemic stroke increases substantially with age, making it the third leading cause of death and the leading cause of long-term disability. With age, women are more likely to suffer stroke and to have more severe stroke outcomes compared to men in the same age group [1]. However, tissue Plasminogen Activator (t-PA), the only currently FDA-approved therapy, is prescribed at lower rates to women than to men stroke patients [2]. Moreover, current opinion supports the need for combining established thrombolytic therapy with effective neuroprotective therapy.

Non-coding RNAs, such as microRNAs (miRNAs) that function as translational repressors are an important regulatory element in both tissue development and disease [3]–[5]. MiRNAs are dysregulated in neurological disorders including stroke and potential therapeutic applications of miRNA have been reported in peripheral and central diseases [6], [7]. Recent studies have identified stroke-induced miRNA in brain and plasma from experimental models and patients [8]–[10]. Additionally, miR120 is positively correlated with better prognosis in stroke patients [11]. Furthermore, antagonists to miR497, infused prior to stroke, reduce infarct volume [12]. However, to date, no neuroprotective miRNA mimics or antagomirs have been identified that are effective when delivered post-stroke.

To identify neuroprotective miRNAs, we used an alternate strategy by interrogating the 3′ UTR of a known neuroprotectant, Insulin-like Growth Factor (IGF)-1, for specific miRNA target sites, with the goal of inhibiting these miRNA to elevate local levels of IGF-1 post-stroke. Insulin-like growth factor (IGF)-1 is a critical endogenous neuroprotectant and low normal levels of peptide hormone are associated with increased morbidity and mortality in ischemic heart disease and stroke [13]–[15]. Exogenous IGF-1 reduces ischemic injury in many species [16]–[19], stimulates stroke-induced neurogenesis [20] and promotes neuronal survival, neuronal myelination and angiogenesis [21]–[22].

Age-related decline in IGF-1 is seen in virtually every species [23] and in middle-aged female rats, loss of IGF-1 is paralleled by declining levels of ovarian hormones such as estrogen. Estrogen is also neuroprotective for stroke [24]–[26] and IGF-1 and estrogen interact to promote neuroprotection in stroke models [27], [28]. The dual loss of these hormones may underlie the more severe infarction seen in middle aged females as compared to young females [29]–[30]. Our studies show that IGF-1 is neuroprotective to middle aged females even when administered 4 h post stroke, making this an attractive candidate for stroke therapy. IGF-1 therapy is also tolerated well in human patients [31], however its capacity to promote tumor (including glioblastoma) growth and metastasis [32], [33], necessitates closely monitoring of its usage especially among older frail populations. Here we report that antagomirs to two miRNAs, miR1 and Let7f, with consensus binding sites in the 3′ UTRs of multiple IGF signaling pathway components confer neuroprotection, while antagomir to a brain-specific miRNA not associated with IGF signaling, was not neuroprotective.

## Results

### Selection of therapeutic miRNA mimetics of IGF

**Figure 1:**
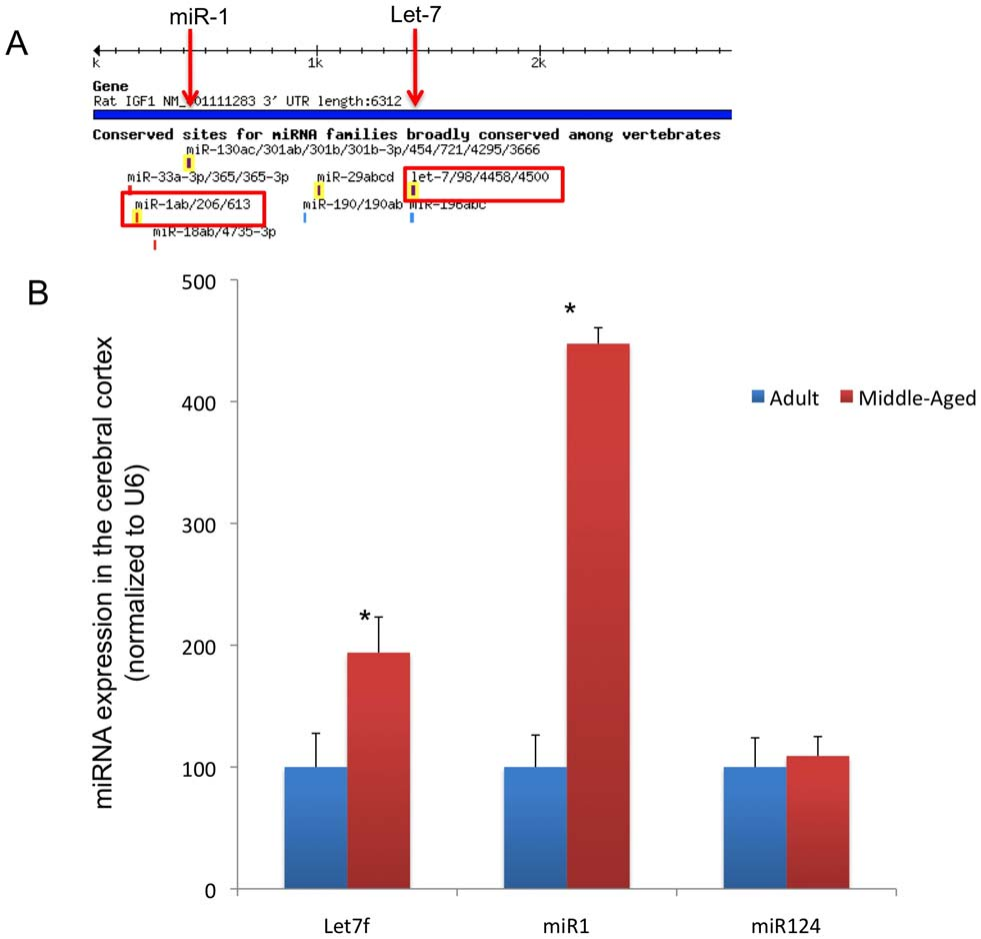
Age-related changes in the expression of selected miRNA associated with the IGF-1 gene. A. Schematic depiction of the 3′UTR of the IGF-1 gene: miR1 and Let7 have preferentially conserved, 7–8 mer and 8 mer binding sites respectively on the 3′UTR region of IGF-1 gene. Nucleotides (nn) indicate the seed region for each microRNA. B. QPCR analyses of microRNA in the cerebral cortex of adult and middle aged females. Expression of miR1 and Let7f is elevated in middle-aged (10–12 month) females as compared to adults females (5–7 month), while age does not affect the expression of miR124, a brain-specific miRNA not associated with IGF-1. *: p<0.05.

We first identified by literature search and *in silico* analysis, two microRNAs, miR1 and the Let7 family as candidate regulators of IGF-1 signaling. Shown in Figure 1A are *in silico* predictions (Targetscan.org; [34]) for vertebrate-conserved target sites for miR1 and Let7f binding in the 3′ UTR of the rat IGF1 gene. Analysis of the published peer-review literature [35]–[42] as well as *in silico* bioinformatics analysis (using several prediction algorithms, Targetscan; www.targetscan.org, miRWalk; http://www.ma.uni-heidelberg.de/apps/zmf/mirwalk, miRDB; http://mirdb.org/miRDB/, and miRanda; http://www.microrna.org) indicates that miR1 and the Let7 family are both functional antagonists of IGFs, and Let7 potentially targets multiple components of the IGF signaling pathway including IGF1 and 2, IGF1R, the IGF mRNA binding and translation regulatory proteins, IGF2BP1, 2 and 3 and the IRS2, a signaling intermediary that couples IGF receptors to intracellular signaling pathways. These analyses indicate that suppressing miR1 and Let7 is both predicted and demonstrated to promote IGF function in a variety of biological systems. In contrast, both published literature and bioinformatics analysis did not implicate miR124, a brain-specific miRNA, in IGF signaling. PCR analysis of adult and middle-aged cortical tissue indicated that expression of both miR1 and Let7f was elevated in middle-aged females (10–12 m old) where IGF-1 levels are reduced, as compared to adult females (5–7 m old) where IGF-1 levels are higher (Fig. 1B). MiR124 expression was not altered with age. In the following experiments, we therefore compared the effects of suppressing miR1 and Let7f (as a prototype member of the Let7 family, conserved throughout vertebrate evolution, [43]), with the effects of suppressing miR124 on functional recovery from stroke.

### Effect of miRNA knock-down on infarct volume in female rats

**Figure 2:**
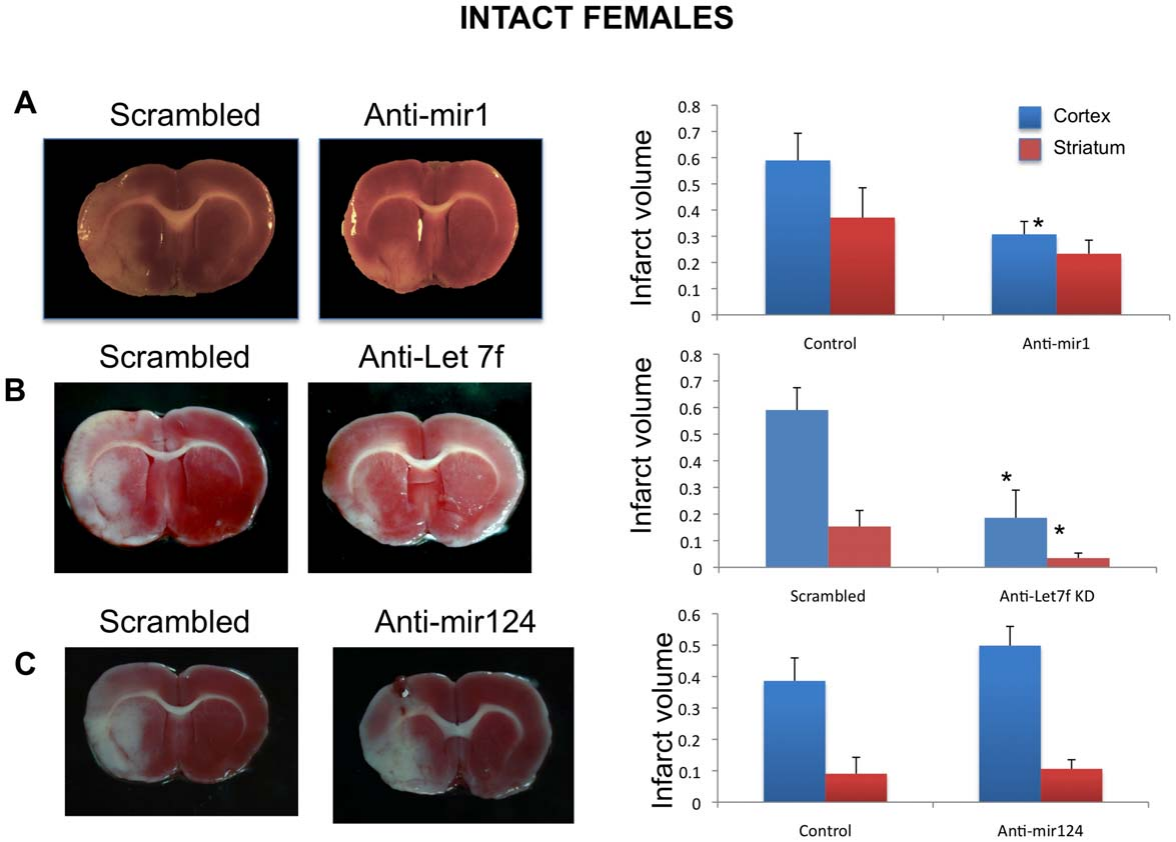
Effect of anti-sense miRNA on infarct volume. Female rats were injected with either anti-sense miRNA or scrambled oligonucleotides (control) 4 h after ET-1 induced middle cerebral artery occlusion (MCAo). Infarct volume was assessed using TTC-stained brain sections and quantitative morphometry. A. Animals injected with anti-miR1 had significantly lower cortical infarct volume as compared to controls injected with a scrambled oligo. Striatal infarct was not significantly different between the two groups. B. Animals injected with anti-Let7f had significantly reduced cortical and striatal infarct volumes as compared to controls. C. Animals injected with anti-miR124 had infarct volumes that were similar to the control group. Histograms depict mean ± SEM. *: p<0.05, n = 5 per group.

Adult female rats were subject to ischemia/reperfusion using endothelin (ET)-1 induced Middle Cerebral Artery occlusion (MCAo). ET-1 delivered to the middle cerebral artery causes a cerebral cortical and striatal infarct typical of other MCAo models [44], [29], [30]. Four hours after ET-1 injection, animals received an intracerebroventricular (ICV) injection of either scrambled oligonucleotides, anti-miR1, anti-Let7f, or anti-miR124 oligonucleotides (LNA-modified, Exiqon, Vedbaek, Denmark). Triphenyl tetrazolium chloride (TTC)-stained brain slices from animals in each group were analyzed for infarct volume. As shown in Figure 2A, cortical infarct volume was significantly reduced (48%, F_(1,12)_: 7.182; p<0.05) by ICV injections of anti-miR1, as compared to animals that received the control (scrambled oligonucleotides). Striatal infarct volume was not significantly altered by miR1 antagomir treatment. In contrast, Let7f antagomirs profoundly reduced both cortical and striatal infarct volume by 69.5% (F_(1,8)_: 57.927; p<0.05) and 77.4% (F_(1,8)_: 26.58; p<0.05) respectively, as compared to the control group (Fig. 2B). As an additional control, we also tested miR124, which has no predicted binding site on the 3′ UTR of the IGF-1 gene or in other IGF signaling components, but is known to promote neuronal differentiation [45]. Antagomirs to miR124 did not affect either cortical or striatal infarct volume (Fig. 2C). In view of the robust, region-independent effect of Let7f antagomirs, subsequent studies focused specifically on this miRNA.

### Sex difference in Let7f effects

**Figure 3:**
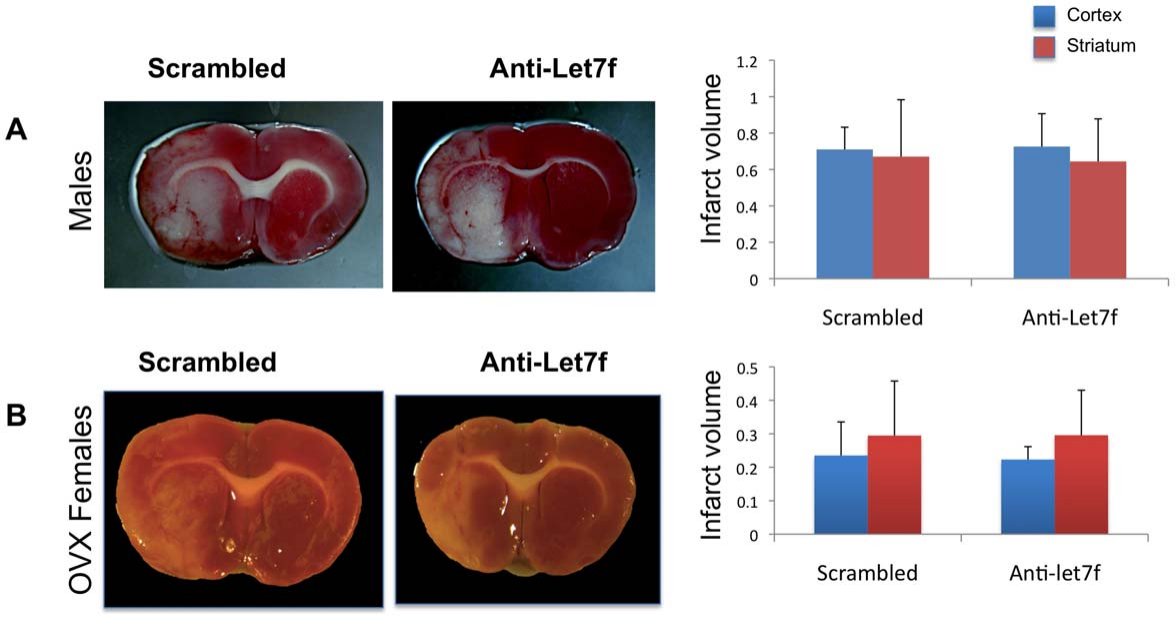
Sex difference in anti-Let7f treatment on infarct volume. A. Male rats received ICV injections of anti-Let7f 4 h post MCAo. Infarct volumes assessed using TTC stained brain sections indicated that anti-Let7f treatment had no effect on the severity of cortical or striatal infarction. B. Ovariectomized female rats received ICV injections of anti-Let7f 4 h post MCAo. Infarct volumes assessed using TTC-stained brain sections indicated that anti-Let7f treatment had no effect on cortical or striatal infarction. Histograms depict mean ± SEM, n = 4–6 per group.

We next determined whether Let7f was also neuroprotective in males. As shown in Figure 3A, cortical or striatal infarct volume was no different between male rats administered control (scrambled) oligonucleotides and anti-Let7f treatment. To determine if the hormonal status of females contributed to the neuroprotective effects of anti-Let7f, females were ovariectomized 3 weeks prior to ET-induced MCAo, to deplete endogenous levels of ovarian hormones. As shown in Figure 3B, ovariectomy resulted in loss of the protective effect of Let7f. The resulting infarct volume was no different in ovariectomized females administered anti-Let7f 4 h post stroke as compared to animals that received scrambled oligonuleotides.

### Behavioral assays

**Figure 4:**
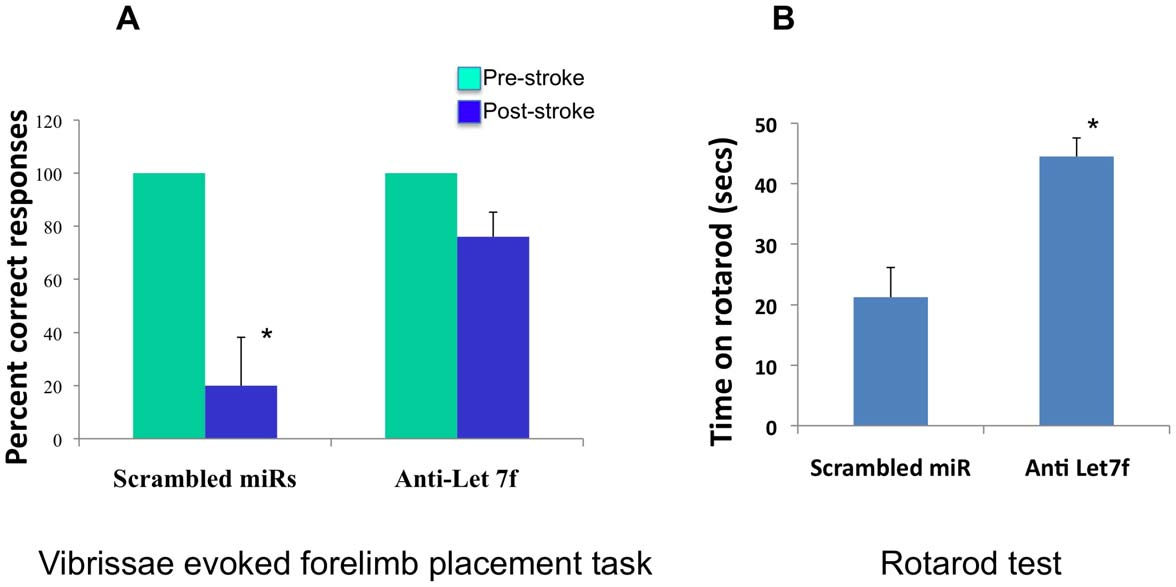
Behavioral assessment of post-stroke anti-Let7f treatment. Animals injected with anti-Let7f or scrambled oligonucleotides 4 h post MCAo were assessed on the vibrissae evoked forelimb placement task and the rotarod task. A. Control animals were impaired post stroke (blue bars) on the cross midline task when the left vibrissa was stimulated (and required right paw placement), as compared to their pre-stroke (green bars) performance. Anti-Let7f treated animals performed no differently from their pre-stroke performance indicating significant functional neuroprotection. Histogram depicts percent correct responses+SEM, N = 4–5/group, *: p<0.05. B. Animals were placed on a slowly-accelerating rod and the amount of time spent on the rod was recorded. Post-stroke, anti-Let7f treated animals were able to maintain their balance on the rotating rod for significantly longer than control treated animals. Histogram depicts time in seconds+SEM, N = 5/group, *: p<0.05.

Anti-Let7f treatment to intact females also improved sensory motor performance. As reported previously, MCAo impairs performance on the vibrissae evoked forelimb placement test [29], [30]. On the “same-side” placement task, where the animal is expected to reach out with the paw located on the same side as the stimulated vibrissae, both control and anti-Let7f treated animals were impaired post stroke (correct responses between 0–10%). This is typical of all cortical-striatal infarcts and is relatively insensitive to the severity or extent of tissue damage. On the “cross midline” placement task, where the animal is expected to reach out with the paw contralateral to the stimulated vibrissae, there was a severe loss of right and left forelimb placement in the control group post-stroke as compared to pre-stroke trials. However, in animals treated with anti-Let7f there was no significant difference between their pre- and post-stroke performance (p>0.05, Fig. 4A), indicating that inter-hemispheric integration of forelimb placement ability was maintained in anti-Let7f treated animals. Integrated sensorimotor function was also preserved in the anti-Let7f treated animals as measured by time spent on the rotarod. Post-stroke, anti-Let7f treated animals spent almost 2 fold more time on the rotating rod as compared to the control group (p<0.05, Fig. 4B).

### Let7f regulation of target gene expression

**Figure 5:**
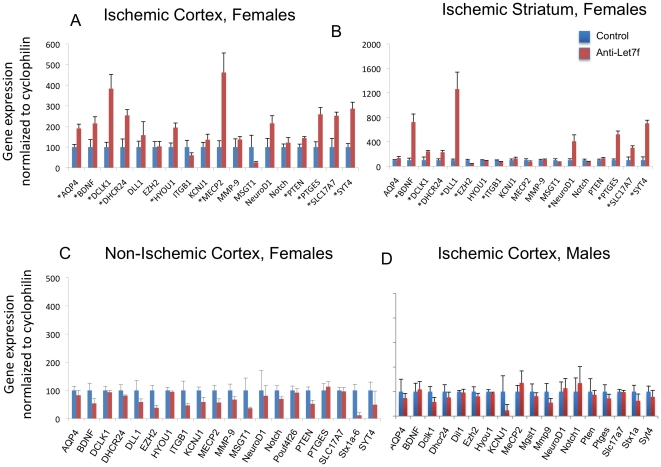
Anti-Let7f affects gene expression in the ischemic brain. RNA from the ischemic cortex and striatum was subject to quantitative PCR for a subset of genes with validated 3′ UTR binding sites for Let7f. Histograms represent gene expression normalized to cyclophilin (control) expression in the cortex (A) and striatum (B). Approximately 10 genes were upregulated in animals that received anti-Let7f as compared to the scrambled controls, indicating that the antagomir inhibited the actions of the endogenous miRNA. An asterisk (located by the gene name) indicates genes that were significantly upregulated by anti-Let7f. A large subset (7/10) of these genes were upregulated by anti-Let7f in both the cortex and striatum, while the remainder (3/10 in each case) were specific to either the striatum or cortex. (C) QPCR analysis of mRNA from the non-ischemic cortex of anti-Let7 and control treated ischemic animals showed no changes in any of the target genes. (D) The same set of genes was also assessed in males treated with anti-Let7f. None of these genes were significantly altered as compared to animals that received scrambled oligonucleotides, consistent with the data that anti-Let7f was not neuroprotective in males. Primers and protein names can be found in Table S2. *: p<0.05.

To determine whether ICV injections of anti-Let7f treatment resulted in local upregulation of Let7f target genes, we used miRWalk, a comprehensive database that provides predicted and validated microRNA targets, to identify a cluster of Let7f target genes. Genes that play a significant role in neuronal survival and/or function were selected for analysis. Of the 18 genes tested, 10 genes were significantly upregulated (p<0.05) in the ischemic cortex of anti-Let7f treated animals (Fig. 5A). These include the growth factor BDNF, the water channel protein Aquaporin-4, enzymes related to sterol (DHCR24) and prostaglandin E2 synthesis (PTGES), genes related to synapse formation (syt4 and MECP2), axonal transport (DCLK1) and cell survival (HYOU1). Gene expression was also elevated in the striatum of anti-Let7f treated animals in comparison to the scrambled controls (Fig. 5B), some of these were similar to the ones seen in the cortex (BDNF, PTGES, SYT4, SLC17A7) as well as genes coding for transcriptional repressor (EZH2), transcription factor (NeuroD1) and integrin B1 (ITGB1). None of these genes were upregulated in the non-ischemic cortex (Fig. 5C). Furthermore, none of these genes were upregulated in male animals subject to stroke followed by anti-Let7f treatment (Fig. 5D).

### Let 7f localization

**Figure 6:**
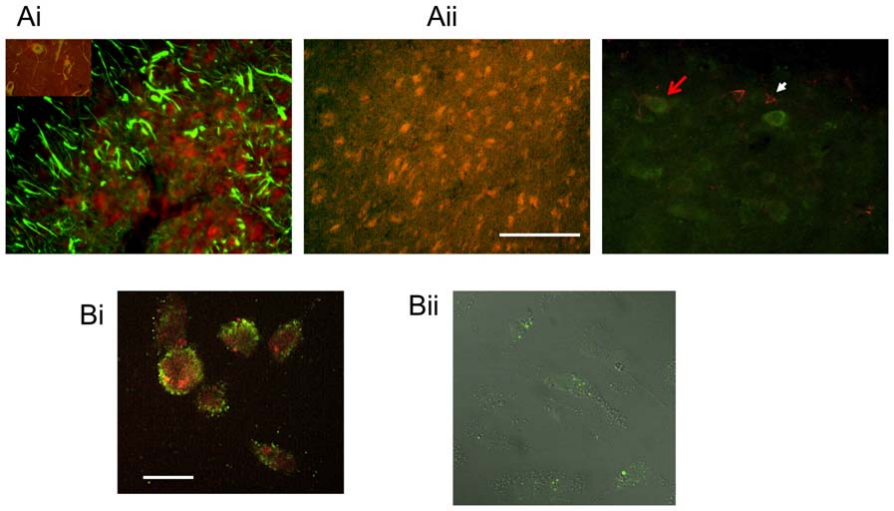
Cellular localization of Let7f in vivo and ex vivo. A. Sections (40 um) from the ischemic cortex were probed for Let7f by in situ hybridization (red) and either GFAP (green, Ai) or CD11b (green, Aii) immunohistochemistry. While Let7f was occasionally colocalized to GFAP-positive cells, virtually all CD11b-positive cells were also Let7f labeled. In a parallel analysis, Let7f (green) was also localized to cells expressing moderate levels of Iba1 (red; red arrow), but not in cells expressing high levels of Iba1 (white arrow). Bar: 200 µm. B. Microglial cells were harvested from the ischemic cortex and cultured *ex vivo* for 2–4 days. (Bi) Combined *in situ* hybridization and immunohistochemistry indicated that all CD11b-positive cells were also positively labeled with Let7f probes. (Bii) Microglial cultures exposed to FAM-labeled anti-Let7f in media readily incorporated the antagomir. Shown here are confocal images of microglia (DIC illumination) with FAM-labeled antagomirs (green) Bar: 30 µm.

In situ hybridization for Let7f combined with immunohistochemistry indicated that Let7f was colocalized occasionally with GFAP-positive cells (astrocytes, Figure 6A, i), but most prominently with CD11b-positive cells (Figure 6A, ii). Since CD11b is not an exclusive marker for microglia, sections were further analyzed for Iba1, a marker for activated microglia. Cells that were strongly labeled for Iba1 had a ramified profile and did not express Let7f, however cells that expressed low to moderate levels of Iba1 co-localized Let7f (Figure 6A, iii). To ensure that Let7f expression in microglia was not due to microglial phagocytosis, we further assessed miRNA expression in these cells *ex vivo*. Microglia were harvested from the ischemic and non-ischemic hemispheres and maintained in culture for 2–4 days. Cultured cells were probed for Let7f and CD11b using double in situ hybridization and immunohistochemistry. As seen in Figure 6B, i, Let7f miRNA *in situ* labeling was seen in all CD11b-positive cells. Additionally, microglial cultures exposed to FAM-labeled anti-Let7f oligonucleotides readily incorporated the oligonucleotide (Figure 6B, ii), indicating that this cell type is likely the target of the anti-Let7f treatments *in vivo*.

### Anti-Let7f increases microglial IGF-1 expression

**Figure 7:**
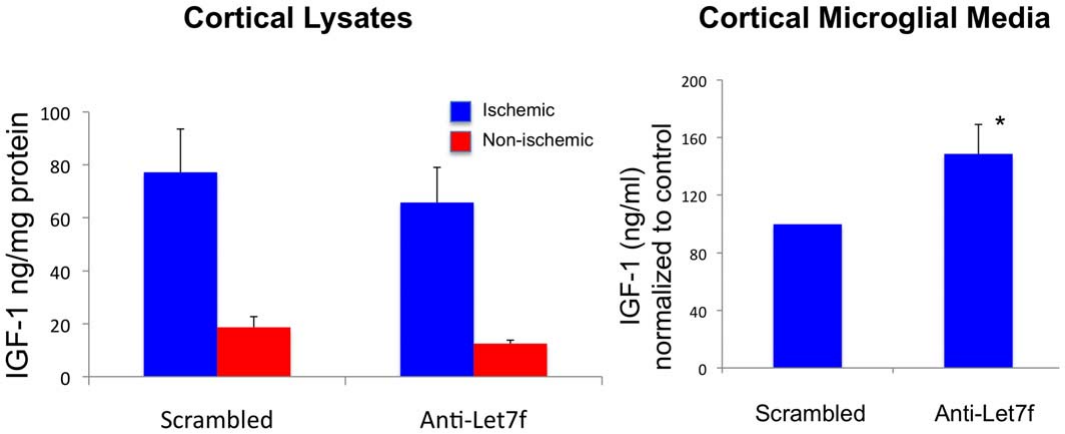
Anti-Let7f regulates IGF-1 in microglia. A. IGF-1 levels in vivo: Cortical lysates from the ischemic and non ischemic hemisphere of scrambled and anti-Let7f treated animals were analyzed for IGF-1 by ELISA. Ischemia induced a 4-fold elevation of IGF-1 in both scrambled and anti-Let7f treated animals. However, IGF-1 levels in the anti-Let7f treated group was no different from that of the scramble controls. Histogram depicts mean ± SEM of 4–5 animals per group. B. IGF-1 levels in microglia: Microglia were harvested from the ischemic cortex of adult rats and treated in culture with anti-Let7f or scrambled oligonucleotides for 48 h. Anti-Let7f treatment significantly increased IGF-1 levels in microglial media. Ex vivo experiments were performed 6 times using cells harvested from 1–2 animals for each run; histogram depicts mean ± SEM of all experiments. *: p<0.05.

Ischemic and non-ischemic cortical tissue from scrambled and anti-Let7f infused animals was analyzed for IGF-1. As shown in Figure 7A, IGF-1 expression was elevated in the ischemic cortex as compared to the non-ischemic cortex, however, anti-Let7f treated animals had IGF-1 levels similar to those that received scrambled oligonucleotides. We next examined IGF-1 levels in microglia exposed to anti-Let7f or scrambled oligonucleotides. Microglia harvested from the ischemic cortex were exposed to antiLet7f or scrambled oligonucleotides (0.25 µM) for 48 h and the media analyzed for IGF-1. As shown in Figure 7B, anti-Let7f significantly increased IGF-1 levels by 48.5% (p<0.05) as compared to cells exposed to scrambled oligonucleotides.

## Discussion

The present study provides novel *in vivo* evidence for a therapeutic role for antagonists to Let7f and miR1 following cerebral ischemia. We [30], and other research groups [17], [46] previously showed that IGF-1 microinjections protected the brain from neurodegeneration following stroke. In the studies reported here, we set out to identify miRNA mimetics of IGF neuroprotection. Our two presumptive IGF pathway interacting miRNAs, Let-7 and miR1 as well as our control, miR124, represent three members of a small family of five miRNAs that have been conserved throughout bilaterian evolution (from invertebrates to mammals) [47], and the functions of the Let7 family, in particular, exhibit strong evolutionary conservation [43]. Accordingly, we reasoned that such highly-conserved miRNAs co-evolved stable networks of regulated gene targets, and that our data from rodent models was likely to be easily extrapolated to human disease. Both *in silico* analyses using a variety of prediction algorithms, as well a large body of experimental literature [35]–[42] indicated that Let-7 and miR1 target multiple components of the IGF signaling cascade, ranging from mRNA binding proteins that coordinate the translation of IGFs to members of the IGF family, their receptors and downstream signaling pathways. Furthermore, both Let7f and miR1 were elevated in middle-aged animals, where IGF-1 levels are low. We therefore hypothesized that the suppression of either Let7 or miR1 would recruit the activation of an evolutionarily conserved network of genes to mimic IGF neuroprotection.

Our data indicate that anti-Let7f (a prototypic vertebrate-conserved member of the Let7 family [43] or anti-miR1, administered four hours following a stroke episode, is indeed neuroprotective to mature female rats. Interestingly, while anti-miR1 reduced infarct volume in the cortex, anti-Let7f reduced infarct volume in both cortex and striatum. This generalized neuroprotection exhibited by anti-Let7f exceeds that exhibited by IGF-1 in female rats [30]. Like miR1, IGF1's neuroprotection is limited to the cortex. Therefore, the neuroprotection in both cortex and striatum afforded by anti-Let7f suggests the recruitment of mechanisms beyond those engaged by IGF1. Moreover, anti-Let7f was overall more effective in inducing neuroprotection following stroke compared to anti-miR1 suggesting that the Let7 family is likely to constitute a stronger therapeutic target for interventional strategies following stroke. Importantly, anti-Let7-mediated tissue preservation was also accompanied by preservation of sensory motor function including performance on the rotorod test and on the contra-lateral vibrissae-induced forelimb placement test. This latter test measures interhemispheric integration of sensory motor information and anti-Let7f mediated preservation of performance on this test following stroke indicates a preservation of integrative neuronal pathways like those projecting through the corpus callosum.

The present study also uncovers a sex difference in stroke-related therapy. Unlike females, anti-Let7f treatment to age-matched male rats had no neuroprotective effect. Sex differences in post-stroke outcome can be influenced by the immediate presence of sex hormones or prenatal hormone exposure or sex chromosomes [48]. Sex differences have been noted in ischemia-induced cell death pathways as well as the effectiveness of neuroprotective compounds. For example, caspase activation is enhanced in ischemic females and caspase inhibition benefits females but not males in neonatal hypoxia [49] and adult ischemia [50]. In contrast, reducing nitric oxide and inhibiting the DNA polymerase PARP1 is neuroprotective for males but deleterious for females [51], [52]. Sex differences in cell death pathways and neuroprotection may be related to the hormonal environment, although the sex chromosomes may also play an important role. In the present study, ovariectomy abolished the protective effect of anti-Let7f in females, suggesting that miRNA action is influenced by the hormonal milieu. In fact, several validated target genes for Let-7f are also regulated by estrogen. A partial list includes the neurotrophin BDNF, the water channel Aquaporin-4 (AQP4), the tumor suppressor gene PTEN, the vesicular glutamate transporter SLC17A7 and the sterol reductase DHCR24 which also functions as a H_2_O_2_ scavenger.

Although Let7f has a consensus binding site on the IGF-1 3′UTR, ICV infusions of anti-Let7f did not elevate IGF-1 mRNA or protein levels in ischemic tissue when measured 5 days post-stroke. IGF-1 protein was elevated on the ischemic hemisphere irrespective of miRNA treatment, consistent with our previous data [30]. One possible explanation is that IGF-1 accumulation at the ischemic site is due to influx of peripheral (plasma) IGF-1, gaining access to the brain due to a stroke-weakened blood brain barrier. Another possibility is that that IGF-1 is regulated by the anti-miRNA treatment at a time point not captured by the current analysis. A more likely explanation, however, is that anti-Let7f acts on a discrete cell type to alter local, but not global, availability of IGF-1. This idea is supported by evidence that anti-Let7f treatment increased IGF-1 in microglia, harvested from the ischemic cortex 2d post-stroke. Microglia are the main effectors of the innate response in the ischemic brain, however their role in post-stroke pathogenesis is widely debated. Microglia release cytotoxic molecules and attenuation of microglial activation promotes neuroprotection [53]. However, microglia also synthesize IGF-1 at the ischemic site [54] and also respond to this peptide by activation of PI-3K and Akt pathways. Furthermore, exogenous application of microglia reduces ischemic injury [55] while selective ablation of proliferating microglia exacerbates ischemic injury and concomitantly reduces IGF-1 levels in the ischemic hemisphere [56]. Microglia play a role in adult neurogenesis [57]–[60] and proliferating microglia serve as an endogenous pool for neurotrophic molecules. Hence, anti-Let7f action on microglia may promote an alternate activation of this phagocytic cell resulting in neuroprotection rather than inflammation. Recent studies have recently shown that even in their resting state microglia interact with neurons and astrocytes [61]–[62] and make direct connections with neuronal synapses [63]. Hence, through its actions on microglia, anti-Let7f may influence other cell types to activate a wide array of genes.

While miRNA act as translational repressors, recent studies indicate that target mRNA are also regulated, suggesting that miRNA may act to destabilize or degrade RNA transcripts [64]. Hence, we examined other Let7f targets to determine the efficacy of anti- Let7f action, focusing specifically on those genes involved in neuronal survival. Anti-Let7f treatment upregulated 55% of these selected mRNAs in the cortex and striatum as compared to controls. Not all the tested genes were elevated by Let7f antagomirs, reflecting the likelihood that Let7 is one of several gene transcription/translation regulators that cooperatively and dynamically alter gene expression following stroke, or alternatively, adverse signal to noise ratio due to the fact that these genes are expressed by small subsets of cells. It does however indicate that gene expression was not simply a reflection of the extent of neuroprotection, in which case all genes would be expected to be elevated. Moreover, none of these genes were upregulated by anti-Let7f in males, consistent with the fact that the antagomir did not improve infarct volume in this sex. The loss of ovarian hormones also rendered anti-Let7f treatment ineffective, suggesting that the sex difference in anti-Let7f treatment is at least partly modified by sex steroids. In fact, the ovarian hormone estrogen has been shown to increase Let7f expression but suppresses miR1 [65], raising the possibility that in intact animals in this study, Let7f antagomir treatment may counteract estrogen-induced Let7f expression and complement estrogen suppression of miR1.

In conclusion, a rapidly growing body of literature on miRNA's role in cancer [66], neurodegenerative diseases [67]–[72] and mental illness [73] strongly supports a therapeutic role for miRNA. Manipulating endogenous miRNA levels by viral vectors or modified oligoneucleotides to mimic or inhibit miRNA are effective in vivo and in-vitro [74]. The present studies indicate a useful strategy for identification of neuroprotective miRNA using known neuroprotectant proteins as a starting point. Both anti-Let7f and anti-miR1 were neuroprotective when administered post stroke, but to differing extents, indicating that other clusters of target genes regulated by these miRNA may critically determine outcomes. A gene cluster based approach, focusing on genes associated with specific disease pathways, may also be a useful strategy. Finally, our data demonstrate a gender-specific role for miRNA, where anti-Let7f mediated neuroprotection was only seen in ovary-intact females, underscoring the importance of the endocrine environment for miRNA actions.

## Methods

### Animals

All animals were purchased from Harlan Laboratories (IN). Females were purchased as proven breeders with 3 pregnancies (6–7 months, 230–320 g, n = 65), while males (n = 9) were age-matched to females. All animals were maintained in a constant 12-h dark:12-h light cycle in AAALAC-accredited vivarium facilities. Food and water were available *ad libitum*.

### Ethics Statement

All animal procedures were in accordance with NIH and institutional guidelines governing animal welfare. An animal use protocol was approved by the Texas A&M IACUC (Animal Welfare assurance # A-3893-01).

### Stereotaxic surgery

The animals were subjected to stereotaxic surgery to occlude the left middle cerebral artery (MCAo) as reported previously [29] for complete details of this procedure). Briefly, MCA occlusion was induced by microinjecting 3 µl of Endothelin-1 (American Peptide Company INC, CA; 0.5 µl in 1 µl PBS). Four hours post-stroke, animals were administered intracerebroventricular (ICV) injections (ICV; coordinates −1.0 mm anterior posterior, +1.4 mm medial lateral, −3.5 mm relative to the dural surface [75] of either scrambled miR (control), anti-Let 7f, anti-miR1 or an unrelated, anti-miR124 antisense LNA-oligonucleotide sequences, (termed “antagomirs”, Exiqon, Vedbaek, Denmark). In each case, 8 µl (25 µM) of the control or antagomir was microinjected over an 8-minute period. This dose was based on manufacturer recommendation (Exiqon, Vedbaek, Denmark) and a modification of a dose reported for intraparenchymal injections of antagomirs [76] to compensate for dispersion in CSF due to intracerebroventricular injections). Animals were randomly assigned to treatment groups. During surgery, rats were maintained at 37°C throughout and oxygen saturation and respiratory rate were constantly monitored using the Mouse Oximeter (STARR life sciences corp. PA). There were no group differences in oxygen saturation and respiration rate (Table S1). At termination, the brain was rapidly removed and processed for TTC (Triphenyl Tetrazolium Chloride) staining to assess infarct volume and biochemical analysis. All animals were sacrificed on day 5 post-MCAo.

In a separate experiment, intact (uninjured) animals (n = 5) were injected ICV with anti-Let7f oligonucleotides and observed for 5 days. These animals displayed no ill-effects from receiving the antagomir, maintained their normal body weight (273+7.38 gms pre-injection, 273.6+5.18 post injection), and showed no differences on the vibrissae evoked forelimb placement task (100% correct responses pre and post injection).

### Infarct volume

Brain slices (2 mm thick) between −2.00 mm and +4.00 mm from Bregma were incubated in a 2% TTC solution at 37°C for 20 min and later photographed using a Nikon E950 digital camera attached to a dissecting microscope. Infarct volume was determined from digitized images using the Quantity One software package (Bio-Rad CA). Typically 3 such slices were used for analysis. The area of the cortical and striatal infarct was measured separately in all slices in the ischemic and non-ischemic hemisphere. Details of the volume determination are described in [29]–[30]. The volume of the infarct was normalized to the volume of the contralateral (non-occluded) hemisphere. To ensure reliable and consistent detection of the infarct zone, images were digitally converted to black and white and magnified, and all traces performed by the same investigator (AS). Application of the volume algorithm and statistical analysis was performed by a separate investigator, blinded to experimental conditions (FS).

### Behavioral assays

Motor impairment following MCAo was assessed using the vibrissae evoked forelimb placement task as well as the rotarod test. The vibrissae-elicited forelimb placement test [77], [29] was used both before and after the MCAo surgery. Animals were subject to same-side placing trials and cross-midline placing trials elicited by stimulating the ipsi and contra-lesional vibrissae (for a detailed description of the procedure refer to 29). Rotarod test: Motor performance of each rat was tested on the Rotarod apparatus (IITC Life Science, CA) the day before MCAo for 3 trials that were 5 minutes in length (training). The animals were tested post-MCAo at 24 hrs. If the animal fell off the beam before 30 seconds, the animal was given a 5 minute rest period, then placed back on the beam for an additional 2 trials [78]. The length of time spent (in sec.) on the beam was recorded.

### Total RNA extraction

Total RNAs from the ischemic cortex of scrambled and anti-Let7f treated males and females was isolated using Rneasy® kit (QIAGEN, Valencia, CA) and quantified using Agilent 2100 bioanalyzer (Agilent Technologies, Inc. Santa Clara, CA).

### QPCR Analysis of Let7f-sensitive genes

Selected validated target genes for miR-Let-7f were identified from the miRWalk (www.ma.uni-heidelberg.de/apps/zmf/mirwalk/) database. Specifically, genes implicated in neuronal survival and function, were selected for QPCR analysis. SYBR green based RT-PCR using MyiQ ™ single-color RT-PCR detection system (Bio-Rad, Hercules, CA) and gene specific primers sets (Rel Time Primers, PA) were used to quantify the following genes, Ezh2, Dll1, Mecp2, Notch1, Pouf4f2, Pten, Bdnf, Aqp4, Syt4, Ptges, Hyou1, Stx1a, Dclk1, Neurod1, Itgb1, Dhcr24, Slc17a7, Mgst1 and Mmp9. Cyclophilin was used as an internal control. Sequences for all primers are reported in Table S2.

### In-situ hybridization and immunoflorescence

Female rats were euthanized 7d post stroke and the brains were removed following intracardial paraformaldehyde perfusion. Coronal brain sections (35 µm thickness) were incubated in pre-hybridization solution (100% Formamide, 5 M Nacl, 50X Denhart, 1 M DTT, 10 mg/ml tRNA and 100% Triton) at 45°C for 2 h. The sections were incubated overnight at 45°C in hybridization solution containing 2.5 µM LNA-modified Let7f oligonucleotide probe, Exiqon (Vedbaek, Denmark). The sections were washed with 5× SSC at room temperature for 5 min followed by a incubation in formamide wash solution (50% Formamide, 0.1% Tween-20, 1× SSC and DEPC) at 45°C for 30 min. Sections were washed in 0.2× SSC at RT for 30 min and then subject to three 5 min washes with PBS followed by incubation with Rhodamine Avidin or Fluorescein Avidin (1∶500, Vector, Burlingame, CA), for 1 h at RT. Following three PBS washes the sections were incubated in the blocking solution (NGS, triton, RNasin and 4 drops/ml of block of avidin blocking solution) at RT for 1 h and then incubated ON with either anti-GFAP (Sigma, St. Louis, MO), anti-CD11b (1∶100, Serotec, Raleigh, NC) or anti-Iba1 (1∶100; Abcam, San Francisco, CA). Sections were then washed in PBS and incubated with a secondary antibody -goat anti-mouse (1∶500, Molecular probes, Carlsbad, CA) or goat anti-mouse (1∶2000; Grand Island, NY). Finally, the sections were rinsed, counterstained with Hoechst (Polyscience, PA) and coverslipped with ProLong (Invitrogen, CA).

### Microglial cultures

Female rats were subject to MCAo and terminated 48 hrs later. Microglia were harvested using one of two procedures: In the first procedure [79], cortical tissue from the ischemic and non-ischemic hemisphere was dissected out under aseptic conditions and rinsed 5× in dPBS with pennicillin/streptomycin. Thereafter, the tissue was mechanically dissociated in Optimem (99%)/N2 (1%) containing media and chemically in trypsin/EDTA for 20 min at RT under sterile conditions. The cell suspension was centrifuged at 700 rpm for 5 min at 18°C and the resultant cell pellet was resuspended in serum containing media plated on poly-d-lysine coated chambered slides and incubated at 37°C. Cells were re-fed day 4 and 7, day 10 the cells were shaken (600 rpm for 15 min) and the media was collected and centrifuged at 700 rpm, 5 min, 18°C and the cells were re-plated in chamber slides at a density of 50,000/chamber. On day 13, the media was collected and the cells were gently fixed in 2% paraformaldehyde (1 h, room temperature) and later processed for Let7f in situ hybridization and CD11b immunohistochemistry. In other experiments, cultures were exposed to FAM-labeled anti-Let7f (0.5 µM) in media overnight. The following day, media was harvested and centrifuged at 1000 rpm for 20 sec at 4°C and stored at −20°C while the cells were gently fixed as before, and coverslipped with Prolong (Invitrogen, CA).

To maintain the ischemic phenotype, the second procedure employed a rapid cell harvest protocol using CD11b antibodies conjugated with magnetic beads (Miltenyi Biotec, CA) according to the manufacturers protocol. Briefly, cortical tissue was collected as above and subject to mechanical and chemical dissociation. Cell suspensions were then filtered and incubated with anti-myelin antibodies to remove myelin fragments. The partially purified cell suspension was later incubated with anti-CD11b antibodies conjugated to magnetic beads and separated on magnetic columns. The resulting cell suspension was placed in chamber slides (50,000/well) and maintained in culture for 48 h. Oligonucleotide treatments were begun thereafter and 2 days later media was collected and cultures fixed for immunohistochemistry. Media was assayed for IGF-1 using an ELISA kit (Mouse/Rat IGF-1 Quantikine ELISA; R & D Systems, MN) using our previously established procedures (30). Microglial cultures were rinsed and fixed in 2% paraformaldehyde and subject to hybridization with LNA modified Let7f oligonucleotide probe (described above) and immunolabeled for CD11b.

### Statistical Analysis

For infarct analysis, group differences were determined by 2-way ANOVA coded for treatment and brain region by multivariate analysis. For most other studies, group differences were determined by Students t-test; for pre- and post-surgery behavioral tests and microglial culture experiments, group differences were determined by paired t-tests. In all cases, differences were considered statistically significant at p<0.05.

## Supporting Information

Table S1 **Physiological parameters recorded during MCA occlusion surgery.**
(DOCX)

Table S2 **Primer sequences for QPCR analysis.**
(DOC)

## References

[1] Bushnell CD (2008). Stroke in women: risk and prevention throughout the lifespan.. Neurol Clin.

[2] Lisabeth LD, Brown DL, Morgenstern LB (2006). Barriers to intravenous tissue plasminogen activator for acute stroke therapy in women.. Gend Med.

[3] Ambros V (2004). The functions of animal microRNAs.. Nature.

[4] Bartel DP (2004). MicroRNAs: genomics, biogenesis, mechanism, and function.. Cell.

[5] Kloosterman WP, Plasterk RH (2006). The diverse functions of microRNAs in animal development and disease.. Dev Cell.

[6] Soifer HS, Rossi JJ, Saetrom P (2007). MicroRNAs in disease and potential therapeutic applications.. Mol Ther.

[7] Kocerha J, Kauppinen S, Wahlestedt C (2009). microRNAs in CNS disorders.. Neuromolecular Med.

[8] Dharap A, Bowen K, Place R, Li LC, Vemuganti R (2009). Transient focal ischemia induces extensive temporal changes in rat cerebral microRNAome.. J Cereb Blood Flow Metab.

[9] Jeyaseelan K, Lim KY, Armugam A (2008). MicroRNA expression in the blood and brain of rats subjected to transient focal ischemia by middle cerebral artery occlusion.. Stroke.

[10] Liu DZ, Tian Y, Ander BP, Xu H, Stamova BS (2010). Brain and blood microRNA expression profiling of ischemic stroke, intracerebral hemorrhage, and kainate seizures.. J Cereb Blood Flow Metab.

[11] Zeng L, Liu J, Wang Y, Wang L, Weng S (2011). MicroRNA-210 as a novel blood biomarker in acute cerebral ischemia.. Front Biosci (Elite Ed).

[12] Yin KJ, Deng Z, Huang HR, Hamblin M, Xie CQ (2010). miR-497 regulates neuronal death in mouse brain after transient focal cerebral ischemia.. Neurobiol Dis.

[13] Schwab S, Spranger M, Krempien S, Hacke W, Bettendorf M (1997). Plasma insulin-like growth factor I and IGF binding protein 3 levels in patients with acute cerebral ischemic injury.. Stroke.

[14] Laughlin GA, Barrett-Connor E, Criqui MH, Kritz-Silverstein D (2004). The prospective association of serum insulin-like growth factor I (IGF-I) and IGF-binding protein-1 levels with all cause and cardiovascular disease mortality in older adults: the Rancho Bernardo Study.. J Clin Endocrinol Metab.

[15] Johnsen SP, Hundborg HH, Sorensen HT, Orskov H, Tjonneland A (2005). Insulin-like growth factor (IGF) I, -II, and IGF binding protein-3 and risk of ischemic stroke.. J Clin Endocrinol Metab.

[16] Gluckman P, Klempt N, Guan J, Mallard C, Sirimanne E (1992). A role for IGF-1 in the rescue of CNS neurons following hypoxic-ischemic injury.. Biochem Biophys Res Commun.

[17] Liu XF, Fawcett JR, Hanson LR, Frey WH (2004). The window of opportunity for treatment of focal cerebral ischemic damage with noninvasive intranasal insulin-like growth factor-I in rats.. J Stroke Cerebrovasc Dis.

[18] Johnston BM, Mallard EC, Williams CE, Gluckman PD (1996). Insulin-like growth factor-1 is a potent neuronal rescue agent after hypoxic-ischemic injury in fetal lambs.. J Clin Invest.

[19] Guan J, Bennet L, George S, Wu D, Waldvogel HJ (2001). Insulin-like growth factor-1 reduces postischemic white matter injury in fetal sheep.. J Cereb Blood Flow Metab.

[20] Yan YP, Sailor KA, Vemuganti R, Dempsey RJ (2006). Insulin-like growth factor-1 is an endogenous mediator of focal ischemia-induced neural progenitor proliferation.. Eur J Neurosci.

[21] Smith LE (2005). IGF-1 and retinopathy of prematurity in the preterm infant.. Biol Neonate.

[22] Wang JM, Hayashi T, Zhang WR, Sakai K, Shiro Y (2000). Reduction of ischemic brain injury by topical application of insulin-like growth factor-I after transient middle cerebral artery occlusion in rats.. Brain Res.

[23] Bartke A (2008). Impact of reduced insulin-like growth factor-1/insulin signaling on aging in mammals: novel findings.. Aging Cell.

[24] Simpkins JW, Rajakumar G, Zhang YQ, Simpkins CE, Greenwald D (1997). Estrogens may reduce mortality and ischemic damage caused by middle cerebral artery occlusion in the female rat.. J Neurosurg.

[25] Dubal DB, Kashon ML, Pettigrew LC, Ren JM, Finklestein SP (1998). Estradiol protects against ischemic injury.. J Cereb Blood Flow Metab.

[26] Rusa R, Alkayed NJ, Crain BJ, Traystman RJ, Kimes AS (1999). 17beta-estradiol reduces stroke injury in estrogen-deficient female animals.. Stroke.

[27] Schabitz WR, Hoffmann TT, Heiland S, Kollmar R, Bardutzky J (2001). Delayed neuroprotective effect of insulin-like growth factor-i after experimental transient focal cerebral ischemia monitored with mri.. Stroke.

[28] Lin S, Fan LW, Rhodes PG, Cai Z (2009). Intranasal administration of IGF-1 attenuates hypoxic-ischemic brain injury in neonatal rats.. Exp Neurol.

[29] Selvamani A, Sohrabji F (2010a). Reproductive age modulates the impact of focal ischemia on the forebrain as well as the effects of estrogen treatment in female rats.. Neurobiol Aging.

[30] Selvamani A, Sohrabji F (2010b). The neurotoxic effects of estrogen on ischemic stroke in older female rats is associated with age-dependent loss of insulin-like growth factor-1.. J Neurosci.

[31] Borasio GD, Robberecht W, Leigh PN, Emile J, Guiloff RJ (1998). A placebo-controlled trial of insulin-like growth factor-I in amyotrophic lateral sclerosis. European ALS/IGF-I Study Group.. Neurology.

[32] Trojan LA, Kopinski P, Wei MX, Ly A, Glogowska A (2002). IGF-I: from diagnostic to triple-helix gene therapy of solid tumors.. Acta Biochim Pol.

[33] Smith J, Axelrod D, Singh B, Kleinberg D (2011). Prevention of breast cancer: the case for studying inhibition of IGF-1 actions.. Ann Oncol.

[34] Lewis BP, Burge CB, Bartel DP (2005). Conserved seed pairing, often flanked by adenosines, indicates that thousands of human genes are microRNA targets.. Cell.

[35] Elia L, Contu R, Quintavalle M, Varrone F, Chimenti C (2009). Reciprocal regulation of microRNA-1 and insulin-like growth factor-1 signal transduction cascade in cardiac and skeletal muscle in physiological and pathological conditions.. Circulation.

[36] Granjon A, Gustin MP, Rieusset J, Lefai E, Meugnier E (2009). The microRNA signature in response to insulin reveals its implication in the transcriptional action of insulin in human skeletal muscle and the role of a sterol regulatory element-binding protein-1c/myocyte enhancer factor 2C pathway.. Diabetes.

[37] Lu L, Katsaros D, de la Longrais IA, Sochirca O, Yu H (2007). Hypermethylation of let-7a-3 in epithelial ovarian cancer is associated with low insulin-like growth factor-II expression and favorable prognosis.. Cancer Res.

[38] Mongroo PS, Noubissi FK, Cuatrecasas M, Kalabis J, King CE (2011). IMP-1 displays cross-talk with K-Ras and modulates colon cancer cell survival through the novel proapoptotic protein CYFIP2.. Cancer Res.

[39] Reid JG, Nagaraja AK, Lynn FC, Drabek RB, Muzny DM (2008). Mouse let-7 miRNA populations exhibit RNA editing that is constrained in the 5′-seed/cleavage/anchor regions and stabilize predicted mmu-let-7a:mRNA duplexes.. Genome Res.

[40] Shan ZX, Lin QX, Fu YH, Deng CY, Zhou ZL (2009). Upregulated expression of miR-1/miR-206 in a rat model of myocardial infarction.. Biochem Biophys Res Commun.

[41] Yu XY, Song YH, Geng YJ, Lin QX, Shan ZX (2008). Glucose induces apoptosis of cardiomyocytes via microRNA-1 and IGF-1.. Biochem Biophys Res Commun.

[42] Hua Y, Zhang Y, Ren J (2011). IGF-1 Deficiency Resists Cardiac Hypertrophy and Myocardial Contractile Dysfunction: Role of microRNA-1 and microRNA-133a.. J Cell Mol Med.

[43] Roush S, Slack FJ (2008). The let-7 family of microRNAs.. Trends Cell Biol.

[44] Biernaskie J, Corbett D, Peeling J, Wells J, Lei H (2001). A serial MR study of cerebral blood flow changes and lesion development following endothelin-1-induced ischemia in rats.. Magn Reson Med.

[45] Makeyev EV, Zhang J, Carrasco MA, Maniatis T (2007). The MicroRNA miR-124 promotes neuronal differentiation by triggering brain-specific alternative pre-mRNA splicing.. Mol Cell.

[46] Rizk NN, Myatt-Jones J, Rafols J, Dunbar JC (2007). Insulin like growth factor-1 (IGF-1) decreases ischemia-reperfusion induced apoptosis and necrosis in diabetic rats.. Endocrine.

[47] Takane K, Fujishima K, Watanabe Y, Sato A, Saito N (2010). Computational prediction and experimental validation of evolutionarily conserved microRNA target genes in bilaterian animals.. BMC Genomics.

[48] Siegel C, Turtzo C, McCullough LD (2010). Sex differences in cerebral ischemia: possible molecular mechanisms.. J Neurosci Res.

[49] Renolleau S, Fau S, Goyenvalle C, Charriaut-Marlangue C (2007). ‘Sex, neuroprotection, and neonatal ischemia’.. Dev Med Child Neurol.

[50] Liu F, Li Z, Li J, Siegel C, Yuan R (2009). Sex differences in caspase activation after stroke.. Stroke.

[51] Hagberg H, Dammann O, Mallard C, Leviton A (2004). Preconditioning and the developing brain.. Semin Perinatol.

[52] Li J, McCullough LD (2009). Sex differences in minocycline-induced neuroprotection after experimental stroke.. J Cereb Blood Flow Metab.

[53] Colton CA (2009). Heterogeneity of microglial activation in the innate immune response in the brain.. J Neuroimmune Pharmacol.

[54] O'Donnell SL, Frederick TJ, Krady JK, Vannucci SJ, Wood TL (2002). IGF-I and microglia/macrophage proliferation in the ischemic mouse brain.. Glia.

[55] Kitamura Y, Takata K, Inden M, Tsuchiya D, Yanagisawa D (2004). Intracerebroventricular injection of microglia protects against focal brain ischemia.. J Pharmacol Sci.

[56] Lalancette-Hebert M, Gowing G, Simard A, Weng YC, Kriz J (2007). Selective ablation of proliferating microglial cells exacerbates ischemic injury in the brain.. J Neurosci.

[57] Butovsky O, Talpalar AE, Ben-Yaakov K, Schwartz M (2005). Activation of microglia by aggregated beta-amyloid or lipopolysaccharide impairs MHC-II expression and renders them cytotoxic whereas IFN-gamma and IL-4 render them protective.. Mol Cell Neurosci.

[58] Butovsky O, Ziv Y, Schwartz A, Landa G, Talpalar AE (2006). Microglia activated by IL-4 or IFN-gamma differentially induce neurogenesis and oligodendrogenesis from adult stem/progenitor cells.. Mol Cell Neurosci.

[59] Ekdahl CT, Claasen JH, Bonde S, Kokaia Z, Lindvall O (2003). Inflammation is detrimental for neurogenesis in adult brain.. Proc Natl Acad Sci USA.

[60] Monje ML, Toda H, Palmer TD (2003). Inflammatory blockade restores adult hippocampal neurogenesis.. Science.

[61] Davalos D, Grutzendler J, Yang G, Kim JV, Zuo Y (2005). ATP mediates rapid microglial response to local brain injury in vivo.. Nat Neurosci.

[62] Nimmerjahn A, Kirchhoff F, Helmchen F (2005). Resting microglial cells are highly dynamic surveillants of brain parenchyma in vivo.. Science.

[63] Wake H, Moorhouse AJ, Jinno S, Kohsaka S, Nabekura J (2009). Resting microglia directly monitor the functional state of synapses in vivo and determine the fate of ischemic terminals.. J Neurosci.

[64] Grimson A, Farh KK, Johnston WK, Garrett-Engele P, Lim LP (2007). MicroRNA targeting specificity in mammals: determinants beyond seed pairing.. Mol Cell.

[65] Bhat-Nakshatri P, Wang G, Collins NR, Thomson MJ, Geistlinger TR (2009). Estradiol-regulated microRNAs control estradiol response in breast cancer cells.. Nucleic Acids Res.

[66] Budhu A, Ji J, Wang XW (2010). The clinical potential of microRNAs.. J Hematol Oncol.

[67] Wang G, van der Walt JM, Mayhew G, Li YJ, Zuchner S (2008). Variation in the miRNA-433 binding site of FGF20 confers risk for Parkinson disease by overexpression of alpha-synuclein.. Am J Hum Genet.

[68] Hebert SS, Horre K, Nicolai L, Papadopoulou AS, Mandemakers W (2008). Loss of microRNA cluster miR-29a/b-1 in sporadic Alzheimer's disease correlates with increased BACE1/beta-secretase expression.. Proc Natl Acad Sci USA.

[69] Kim J, Inoue K, Ishii J, Vanti WB, Voronov SV (2007). A MicroRNA feedback circuit in midbrain dopamine neurons.. Science.

[70] Wang S, van der Walt JM, Mayhew G, Li YJ, Zuchner S (2008). The endothelial-specific microRNA miR-126 governs vascular integrity and angiogenesis.. Dev Cell.

[71] Johnson R, Zuccato C, Belyaev ND, Guest DJ, Cattaneo E (2008). A microRNA-based gene dysregulation pathway in Huntington's disease.. Neurobiol Dis.

[72] Packer AN, Xing Y, Harper SQ, Jones L, Davidson BL (2008). The bifunctional microRNA miR-9/miR-9* regulates REST and CoREST and is downregulated in Huntington's disease.. J Neurosci.

[73] Beveridge NJ, Tooney PA, Carroll AP, Gardiner E, Bowden N (2008). Dysregulation of miRNA 181b in the temporal cortex in schizophrenia.. Hum Mol Genet.

[74] Hutchison ER, Okun E, Mattson MP (2009). The therapeutic potential of microRNAs in nervous system damage, degeneration, and repair.. Neuromolecular Med.

[75] Paxinos G, Watson C, Pennisi M, Topple A (1985). Bregma, lambda and the interaural midpoint in stereotaxic surgery with rats of different sex, strain and weight.. J Neurosci Methods.

[76] Krützfeldt J, Kuwajima S, Braich R, Rajeev KG, Pena J (2007). Specificity, duplex degradation and subcellular localization of antagomirs.. Nucleic Acids Res.

[77] Woodlee MT, Asseo-Garcia AM, Zhao X, Liu SJ, Jones TA (2005). Testing forelimb placing “across the midline” reveals distinct, lesion-dependent patterns of recovery in rats.. Exp Neurol.

[78] Sayeed I, Wali B, Stein DG (2007). Progesterone inhibits ischemic brain injury in a rat model of permanent middle cerebral artery occlusion.. Restor Neurol Neurosci.

[79] Johnson AB, Sohrabji F (2005). Estrogen's effects on central and circulating immune cells vary with reproductive age.. Neurobiol Aging.

