# Supplementary material for: An Antagomir to MicroRNA Let7f Promotes Neuroprotection in an Ischemic Stroke Model
Source: PLoS One. 2012 Feb 29;7(2):e32662. doi: 10.1371/journal.pone.0032662 (PMC3290559; doi:10.1371/journal.pone.0032662)
Supplement: Table S1 — Physiological parameters recorded during MCA occlusion surgery. (DOCX) [file pone.0032662.s001.docx]

**Supplementary Table S1**

Mean oxygen saturation and respiratory rate recorded during MCA occlusion surgery

|  | Scrambled | | Anti-Let7f | | Anti-Mir1 | | Anti-miR124 | |
| --- | --- | --- | --- | --- | --- | --- | --- | --- |
|  | O2 Sat | Resp Rate | O2 Sat | Resp Rate | O2 Sat | Resp Rate | O2 Sat | Resp Rate |
| Females | 91.57  +1.9 | 70.21  +3.7 | 90.73  +1.1 | 70.37  +4.7 | 91.42  +2.2 | 70.11  +2.5 | 92.21  +1.8 | 71.06  +3.9 |
| Males | 90.97  +1.5 | 70.35  +2.2 | 90.17  +1.1 | 68.03  +3.6 |  |  |  |  |
| OVX females | 90.58  +0.7 | 69.81  +2.3 | 90.42  +1.1 | 69.28  +2.5 |  |  |  |  |

O2 Sat: Oxygen Saturation

Resp Rate: Respiration Rate

OVX: Ovariectomized
