# Supplementary material for: An Antagomir to MicroRNA Let7f Promotes Neuroprotection in an Ischemic Stroke Model
Source: PLoS One. 2012 Feb 29;7(2):e32662. doi: 10.1371/journal.pone.0032662 (PMC3290559; doi:10.1371/journal.pone.0032662)
Supplement: Table S2 — Primer sequences for QPCR analysis. (DOC) [file pone.0032662.s002.doc]

**Supplementary Table S2: Primer sequences**

| **Gene** | **NCBI** | **Protein** | **Forward Primer** | **Reverse Primer** |
| --- | --- | --- | --- | --- |
| Aqp4 | | NM_012825 | | --- | | Aquaporin-4 | 5’ - CCG TAC CCA AGA GAC AGC TA - 3’ | 5’ - AGT GTA TGG ACC ACC TCG AA - 3’ |
| Bdnf | | NM_012513 | | --- | | Brain-derived neurotrophic factor | 5’ - CAT TTC ATG ACA CTC GTG GA - 3’ | 5’ - ATT TCA GTG GCA GTG TGG AT - 3’ |
| Dclk1 | | NM_021584.2 | | --- | | Serine/threonine-protein kinase | 5’ - AAG AAG AGT CCG ACG AAG GT - 3’ | 5’ - CGG ATG CTT CAC TCT CCT TA - 3’ |
| **DHCR24** | NM_001080148.1 | 24-dehydrocholesterol reductase | 5’ – CTT GGT GTC TAT GGG TCA GG | 5’ TGT TGG AAC AGG CCA TAC TT |
| **Dll1** | NM_032063.2 | Notch Delta ligand | 5’ - GGT TCA TCT CGG AGT TAG CA - 3’ | 5’- TGC TTC CTG TGT GAA CTC AA - 3’ |
| **Ezh2** | NM_001134979.1 | Histone-lysine N-methyltransferase | 5’ - GGA GGG AGC TAA GGA GTT TG - 3’ | 5’ - CCC TGC TTC TCT GTC ACT GT - 3’ |
| **Hyou1** | NM_138867 | Hypoxia upregulated protein-1 | 5’ - TGT GTC TTC TCT TCC CTT GC - 3’ | 5’ - TAC AGG GCT TTT CCC TTC TT - 3’ |
| **Itgb1** | NM_017022 | Integrin beta 1 | 5’ - TGT GTG TGC AGG AAG AGA GA - 3’ | 5’ - ATG GAA CAG TGT CCA AGG AA - 3’ |
| **Kcnj16** | NM_017023.1 | Potassium inwardly-rectifying channel, | 5’ - CTG TGA GAA CCC AGA GGA GA - 3’ | 5’ - CCG CTG CAA ATT ACA GAA CT - 3’ |
| **Mecp2** | NM_022673 | Methyl CpG binding protein 2 | 5’ - AGC AGC ATC AGA AGG TGT TC - 3’ | 5’ - CTT CAG CTT TTC GCT TTC TG - 3’ |
| **Mmp9** | NM_031055 | Matrix-metalloproteinase-9 | 5’ - ACT TCT GGC GTG TGA GTT TC - 3’ | 5’ - TGT ATC CGG CAA ACT AGC TC - 3’ |
| **Mgst1** | NM_134349 | Microsomal glutathione S-transferase 1 | 5’ - GAC TGC ATT CCA GAG GCT AA - 3’ | 5’ - CTG AAG TGA ATG AGG GCT GT - 3’ |
| **Neurod1** | NM_019218 | Neurogenic differentiation factor 1 | 5’ - CGA TTA GAG GCA CGT CAG TT - 3’ | 5’ - TTC TTC CAA AGG CAG TAA CG - 3’ |
| **Notch 1** | NM_001105721.1 | Notch homolog 1, translocation-associated | 5’ - AGG AAA CAA CTG CAA GAA CG - 3’ | 5’ - TGT TCT CAC TGC AGT CCT CA - 3’ |
| **Pten** | NM_031606.1 | Phosphatase and tensin homolog | 5’ - GAA AGG ACG GAC TGG TGT AAT - 3’ | 5’ - CTG GTC CTT ACT TCC CCA TAA - 3’ |
| **Ptges** | NM_021583.2 | Prostaglandin synthase | 5’ - GGC TAA GCT AGG TGT GTG GA - 3’ | 5’ - ATA TGG CAT GAT GGC TCT GT - 3’ |
| **Slc17a7** | NM_053859 | Vesicular Glutamate transporter 1 | 5’ - CGT ATC CAT GGT CAA CAA CA - 3’ | 5’ - GCG AAT TTT TGG CAG ATA AA - 3’ |
| **Syt4** | NM_ 031693 | Synaptotagmin-4 | 5’ - CCT GTC AGA AGC CTC AGT GT - 3’ | 5’ - GTG AGG GGG AAA GAC AAT CT - 3’ |
